# Suicidal Ideation, Depression, and Aggression Among Students of Three Universities of Isfahan, Iran in 2008

**Published:** 2012

**Authors:** Seyed Ghafur Mousavi, Kasra Keramatian, Mohammad Reza Maracy, Mehdi Fouladi

**Affiliations:** 1 Asssociate professor of psychiatry, Behavioral Sciences Research center(BSRC), Department of Psychiatry, Isfahan University of Medical Sciences(IUMS),Isfahan,Iran,; 2 Assistant professor of epidemiology, Department of Social Medicine, IUMS.

**Keywords:** Aggression, College Students, Depression, Suicidal Ideation

## Abstract

**Objective:** University students’ mental health affects not only their educational achievements, but also their professional future. The authors assessed the prevalence of suicidal ideation, depression, and aggression among students of three major universities in Isfahan, Iran.

**Methods:** In 2008, 470 students were entered into the study using a convenience sampling method. The three measurement tools applied were Aggression Questionnaire (AGQ), Beck Depression Inventor (BDI), and Beck Scale for Suicide Ideation (BSSI).

**Results:** Suicidal ideation was present in 7.58% of the students, depression in 28.04%, and aggression in 30.11% of them. The ratio of depression to suicidal ideation was approximately 4:1. No significant difference in the mean scores of aggression, depression, and suicidal ideation was observed between the three universities. No significant relationships were found between mean scores of aggression, depression, and suicidal ideation with age and gender. There was no meaningful relationship between the mean scores of aggression and marriage status, but the mean scores of depression (**P** = 0.01) and suicidal ideation (**P **= 0.0001) were significantly lower in the married students compared to the single ones. Aggression was significantly associated with depression and suicidal ideation (P = 0.0001).

**Conclusion:** The frequency of suicidal ideation, aggression, and depression was less in our studied college students than in previous non–Iranian studies. The decreasing trend in reported frequency of mild depression during previous years is a noticeable finding. Yet, the findings seek more preventing programs among college students.

## Introduction

University student life consists of many areas including social interactions with peers, moving away from family and friends, change in financial situation, plus routine educational tasks. Mental health of students is related to some factors such as their coping mechanisms, social supports, and ability to adapt (1). In fact many of college students will take important roles in the future of the society, so taking care of their health, especially mental and spiritual health, is necessary.

Depression, aggression, and suicidal ideation are common psychiatric problems that, separately or in combination, can cause mental, somatic, or socio-economic distress or impairment. Previous studies have shown more aggression in those with suicidal ideations (2) and a significant correlation between history of aggression and suicidal attempts (3). Some studies have also shown the relationship between depression, aggression, and suicidal ideation (1, 2, 4, 5, 6). 

Although some previous studies have reported relatively high frequencies of depression, aggression, and suicidal ideation among the university students and have focused on studying high risk cases (7), little is known about the related situations of factors that precipitate these pathologies and their correlation with specific aspects of student life. Furthermore, we have little information about the frequencies of these pathological traits among the Iranian college students.

So, in this study, we aimed to assess the prevalence of aggression, suicidal ideations, and depression among the students of three major universities in Isfahan, Iran. Also we studied the relationship between these three factors (i.e. suicidal ideation, aggression and depression) and the gender, age, and marriage situation of the students. 

## Materials and Methods

*Study population*

This cross-sectional study was conducted in the second semester of the 2008 academic year (from February to September). There are three major universities in Isfahan: Isfahan University (for economics, laws, education, biology, language and other sciences), Isfahan University of Medical Sciences, and San’ati University of Isfahan (for Engineering and other industrial sciences). At the time of study 28,000 students were studying in these three universities (14500, 5500, and 8000 students, respectively). The sample size was 470 students, considering the confidence interval (CI) of 95%, study power of 90% and 10% of probable missing data. We used convenience stratified sampling; so 245, 90, and 135 questionnaires were given out to students who were studying in these three universities in the considered semester. Those who did not respond to the questionnaires, those who did not answer all the questions and those respondents who chose multiple answers to one question were excluded.

*Measures*

Each questionnaire contained of four self-administered parts:

1. Demographic information: This contained age, gender, marriage situation, and the university studying in.

2. AGQ: This is a full revision of the Buss-Durkee Hostility Inventory, a widely-used measure assessing hostility and aggression. It was published in 1992 by Buss and Perry (8). It has been modified for Iranian subjects and consists of 30 items in which 14 items evaluate anger, eight items evaluate aggression, and eight ones evaluate vindictiveness. All 30 items use a 4-point Likert scale response format, ranging from '0 = Never' to '4 = Always'. Scores for individual respondents were obtained by summing their responses to all the items of the scale. The total score can range from 0 to 90 and the scores more than 45 considered as the presence of aggression (9). 

3. Beck Depression Inventory-Second edition (BDI-II): This questionnaire is used to evaluate the clinical symptoms of depression (10). Modified Persian version of this questionnaire has been evaluated by Ghasemzade et al. and its reliability (Cronbach’s alpha) has been reported as 0.87 (11). This questionnaire consists of 21 items and each of them evaluates one clinical aspect of depression. Each item contains four statements, from 0 (healthy status in that aspect) to 3 (severe and deep problem in that aspect). Total score is from 0 to 63. Scores from 0 to 13 are considered as the absence of depression, 14 to 19 as mild, 20 to 28 as moderate, and 29 to 63 as severe depression.

4. BSSI: BSSI contains 19 items each one consists of three statements scoring from 0 to 2 (12). The first five items evaluate the presence of death wish, seven items evaluate preparation for suicide, five items evaluate desire to suicide, and two items evaluate the inhibition and denial of suicide. The total score can range from 0 to 38. Scores of 0-5 show having suicide ideation (low risk), 6-19 preparation for suicide (high risk), and 20-38 show decision to suicide (very high risk). The validity of this questionnaire has been approved by Anisi et al. for Iranian population and its reliability (Cronbach’s alpha) was found to be 0.95 (13).

*Data collection*

The questionnaires were distributed in public places of the studied universities such as libraries, restaurants, and dormitories. Before filling the questionnaire, the purpose of the study and the method of filling were explained to each student. The questionnaires were filled out anonymously. The meantime taken for its filling was 25 minutes. The response rate was 92.5%.

*Statistical analysis*

AGQ scores were analyzed with t-test and analysis of variance (ANOVA). Because of violation of the normality assumption of parametric tests, Mann-Whitney H and Kruskal-Wallis U tests were used for BDI and BSSI scores. In addition, Pearson's correlation test was used to evaluate the association between the three scores. A P value of less than 0.05 was considered statistically significant.

## Results

Of 470 questionnaires, 435 (92.5%) were filled out completely and included in the study. There were 254 female (58.4%) and 181 (41.6%) male participants with mean (±SD) age of 21.76 (±2.33) years. Among the studied students, 382 (87.8%) were single, 53 (12.2%) were married, and no one was divorced.

Suicide ideation was present in 33 (7.58%) students, from whom 28 (6.43%) were high risk, and 5 (1.14%) were very high risk, based on BSSI categorization. The mean (±SD) score obtained from BSSI questionnaires was 3.89 (±1.68) (the scores were ranging from 0 to 30) and no significant difference was found among the mean scores of BSSI in three universities (Kruskal-Wallis test, P = 0.89).

Some grades of depression were found in 122 (28.04%) students. Mean (±SD) score of BDI was 10.02 (±8.88) (range, 0 to 51). Mild, moderate, and severe depression was observed in 60 (13.79%), 42 (9.65%), and 20 (4.59%) students, respectively. There was no significant difference among the mean scores of BDI in the three universities (Kruskal-Wallis test, P =0.37).

Aggression was reported in 131 (30.11%) students. Mean (±SD) score of AGQ was 38.55 (±11.81) (range, 13 to 82). There was no significant difference among the three studied universities in terms of mean score of AGQ (ANOVA teat with F_ (2,432)_ =0.241, P=0.78)

Mean score of depression showed significant direct correlation with mean scores of suicide ideation and aggression (r=0.536, P<0.0001 and r=0.448, P<0.0001, respectively). In addition, suicide ideation had a significant direct correlation with aggression (r=0.248, P<0.0001). Similar relationships were found while the data were analyzed for each individual university.

Mean score of aggression was not statistically different between single and married students (Kruskal-Wallis test, P =0.48), but mean scores of depression and suicide ideation were significantly higher in single students compared with the married (Kruskal Wallis test; P=0.01 and P=0.0001, respectively) (Table 1).

**Table 1:** Scores of depression, suicidal ideation, and aggression in singles and married students of all the three studied universities of Isfahan, Iran

** Scores of**	** Married **	** Single**	** P value**
	Mean± SD	Mean ±SD	
**Depression**	10.12+_ 7.47	10.37+_ 8.65	0.01
**Suicidal ideation**	3.82 ± 1.23	3.90± 1.74	0.0001
**Aggression**	37.49± 12.63	38.70±11.70	0.48

The correlation between the three studied mean scores (depression, suicide ideation, and aggression mean scores) and students’ age was not statistically significant (r=-0.005, P=0.92; r=0.060, P=0.21; and r=0.084, P=0.07; respectively). Moreover, mean scores of depression, suicide ideation, and aggression were not significantly different in males vs. females (Kruskal-Wallis test; P=0.47, P=0.53, and P=0.66, respectively) (Table 2).

**Table 2:** Scores of depression, suicidal ideation, and aggression in male and female students of the three studied universities of Isfahan, Iran

**Scores of**	**Male**	**Female**	**P value**
	Mean± SD	Mean ±SD	
**Depression**	9.44±8.11	10.43±9.38	0.47
**Suicidal ideation**	4.03± 1.68	3.80±1.68	0.53
**Aggression**	38.22±11.25	38.76±11.25	0.66

## Discussion

The suicidal ideation, depressive symptoms, and aggression were studied in a sample of students from three main universities located in Isfahan, Iran. Suicidal ideation was reported in7.59% of them, aggression in 30.11%, and depression in 28.44%. 

*Suicidal ideation*

In our study, 7.59% of the students had suicidal ideations. A previous study on university students of Isfahan in 2005 reported this figure as 10.3% (14). Some other studies on Chinese and Spanish students reported the prevalence of suicidal ideation as 18% and 34.4%, respectively (15,16). A study on Pakistani University students showed a rate of 31.4% in suicidal ideations (17). Our study showed a noticeable lower frequency of suicidal ideation than the results of non-Iranian studies. Cultural and religious beliefs in our country that prohibits suicide may explain this difference. Yet, we believe that our reported rate is noticeable and more preventive measures seem to be necessary. 

Many studies have reported the higher prevalence of suicidal ideation in women than in men (15,18-20). However, the prevalence of suicidal ideation has been reported to be more prevalent in male students of the universities of Isfahan than in female ones (14), we found no significant relation between sex and suicidal ideation. 

We found that the prevalence of suicidal ideation was significantly lower in married students comparing with single ones. This finding is consistent with the results of previous studies (21-23). These reports support the belief that marriage can improve mental health.

*Depression*

The frequency of depression in our studied students was 28.04%. This rate was reported as 25.7% and 31.7% in two other Iranian studies (24, 25). Similar studies in Hong Kong and the United States reported this rate as 50% and 23%, respectively (26, 27). As such, the prevalence of depression in our study is similar to the previous reports, especially those in Iran. 

We found that 49.18% of the students with depressive symptoms had mild depression. Ahmadi et al. reported this figure as 76.6% (28). The decreasing trend in reported frequency of mild depression during previous years is a noticeable finding. 

As in many other studies, we did not find any meaningful relationship between depression and sex and, also age (24-26, 29) but the prevalence of depression in single students was significantly higher than in married ones. Two previous Iranian studies found the lower frequency of depression in single students than in married ones; however, the differences were not statistically significant in either study (25,28). 

*Aggression*

In this study, 30.11% of the participants found to have some degrees of aggression. Based on the results of many studies, aggression is a common behavior in the youth and its maximum prevalence is observed in those who aged 18-30 years (30-32). In a study in Boston, 44% of female and 16% of male students were found to have some grades of aggression (33). In another study in Switzerland these figures were reported to be 25% and 33% among female and male students, respectively (34).

Unlike many studies that have reported the prevalence of aggression to be higher in men, than in women (34-36), our study did not show any meaningful association between sex and aggression. 

We did not find any significant correlation between aggression and marriage. In many other studies, aggression has been reported to be lower in married individuals than in singles (37, 38).

These findings are somehow in accordance with the Parks et al. model for decreasing suicidal ideation and violent behaviors by improving self esteem and reducing depression (39). 

In brief, the ratio of suicidal ideation to depression was approximately 1:4, and the ratio of suicidal ideation to aggression was 1:3.7 in our study. These ratios may be a base for future studies and intervention for improving the mental health of college students.

## Authors’ Contributions

SGM conceived and designed the evaluation, interpreted the clinical data, supervised different phases of study, and drafted the manuscript. KK collected the clinical data, and helped to draft the manuscript. MRM participated in designing the evaluation and performed the statistical analysis. MF participated in collecting the clinical data. All authors read and approved the final manuscript.

## Notes

**Declaration of interest: **None.
